# Immune‐mediated thrombocytopenia induced with durvalumab after chemoradiotherapy in a non‐small cell lung cancer patient: A case report

**DOI:** 10.1111/1759-7714.14106

**Published:** 2021-08-17

**Authors:** Tomonori Makiguchi, Hisashi Tanaka, Kosuke Kamata, Kageaki Taima, Akira Kurose, Sadatomo Tasaka

**Affiliations:** ^1^ Department of Respiratory Medicine Hirosaki University Graduate School of Medicine Hirosaki Japan; ^2^ Department of Gastroenterology and Hematology Hirosaki University Graduate School of Medicine Hirosaki Japan; ^3^ Department of Pathology Hirosaki University Graduate School of Medicine Hirosaki Japan

**Keywords:** amegakaryocytic thrombocytopenic purpura, concurrent chemoradiotherapy, immune checkpoint inhibitor, immune‐mediated thrombocytopenia

## Abstract

We describe a case of a 60‐year‐old man with a prolonged thrombocytopenia during a durvalumab maintenance therapy after chemoradiotherapy for locally advanced non‐small cell lung carcinoma. Bone marrow specimen was normoplastic with the marked megakaryocyte depletion, which was assumed to be an acquired amegakaryocytic thrombocytopenic purpura. Although hematological disorders as immune‐related adverse events (irAE) are rare, we should pay more attention to hematological disorders with durvalumab especially after concurrent chemoradiotherapy.

## Graphical abstract

We assumed that durvalumab‐induced thrombocytopenia was attributable to amegakaryocytic thrombocytopenic purpura, which is characterized by dysregulated immunity against thrombopoietin or its receptor.

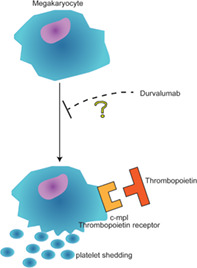

## INTRODUCTION

Immune checkpoint inhibitors (ICIs) have been administered to patients with a variety of cancers and are known to cause specific adverse events (i.e., immune‐related adverse events [irAE]). Compared to other adverse events, including endocrine, dermatological, hepatic, gastrointestinal, or lung toxicity, hematological irAEs are rare, occurring at a frequency of 0.5% for grade 2 or more.^1^, ^2^ Among these hematological irAEs, thrombocytopenia was reported to be the most common type. ICIs are recognized as more tolerable than cytotoxic agents regarding hematological toxicity, whereas fatal immune‐mediated thrombocytopenia has been reported.^3^ Here, we present a case of non‐small cell lung cancer (NSCLC) with a prolonged thrombocytopenia during maintenance therapy with durvalumab following concurrent chemoradiotherapy.

## CASE REPORT

**FIGURE 1:**
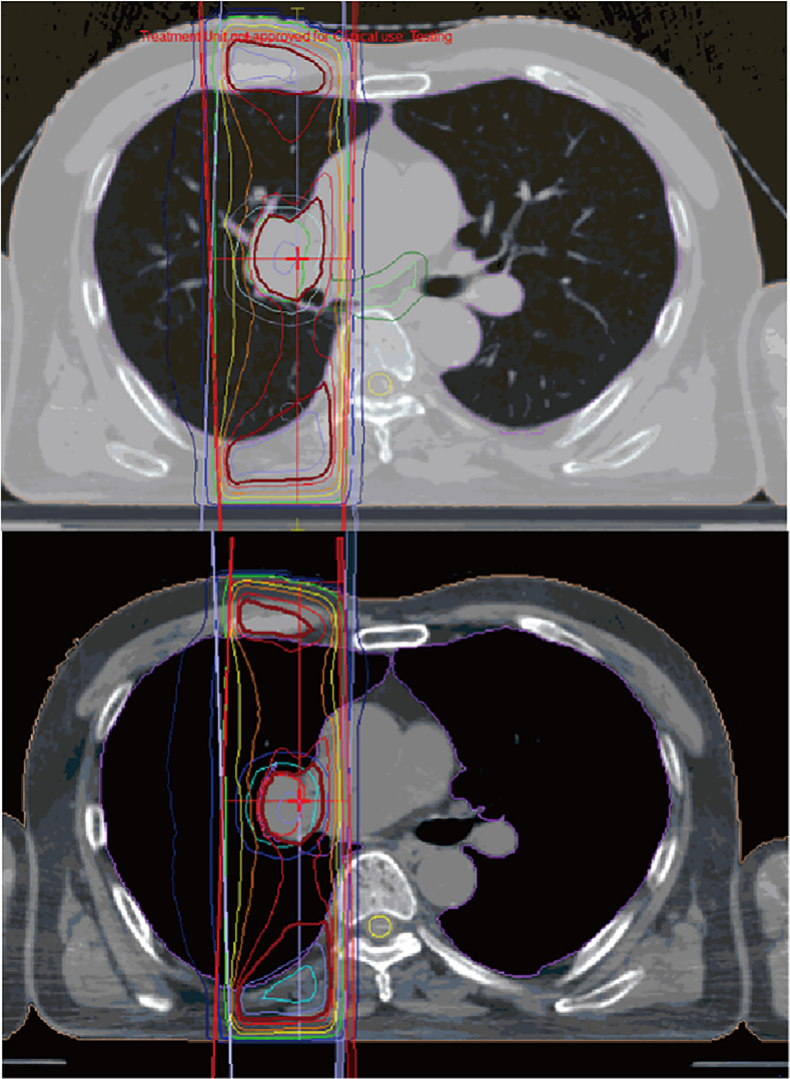
The design of irradiation. The irradiation area was close to the spinal cord

**FIGURE 2:**
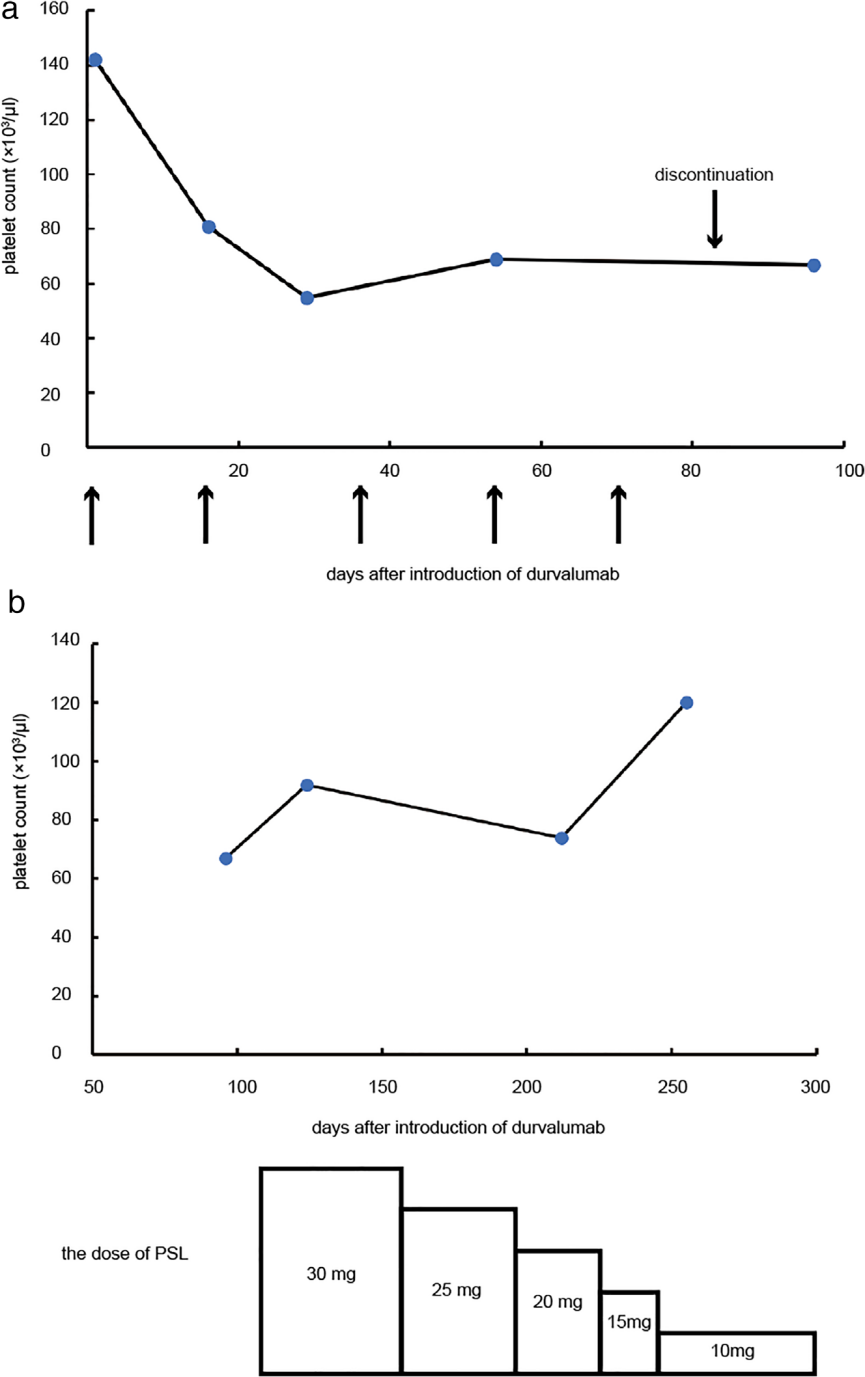
The change in platelet counts following initiation of durvalumab and introduction of prednisolone. (a) Time course of platelet counts from initiation to discontinuation of durvalumab. Arrow represents administration of durvalumab. (b) The change in platelet counts following introduction of prednisolone

**FIGURE 3:**
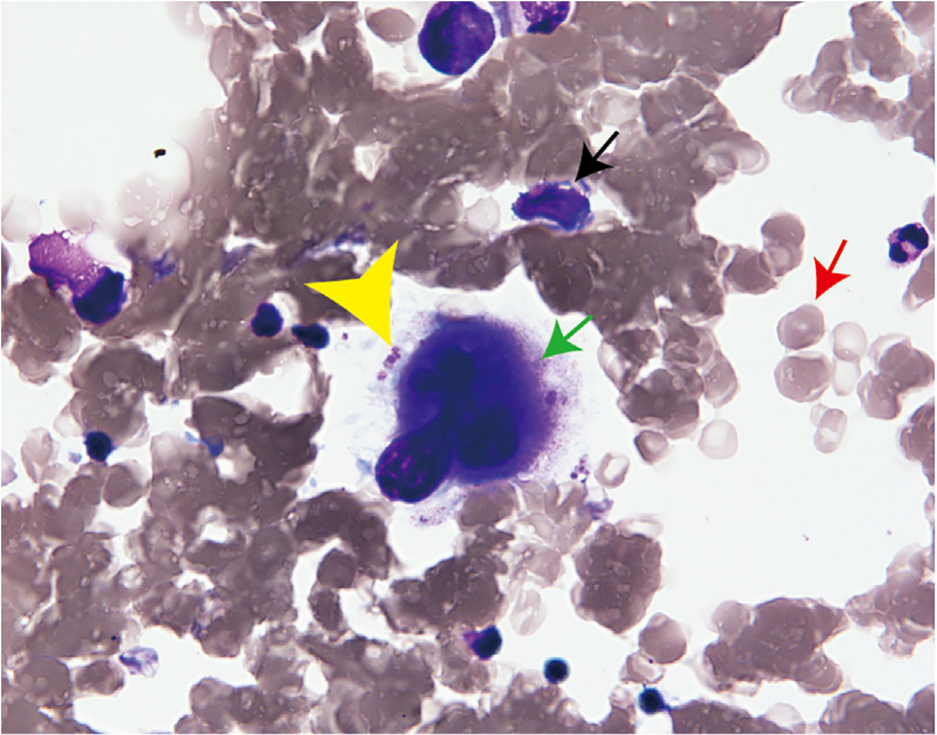
Findings of bone marrow specimen at a high magnification. Bone marrow specimen comprised all the three types of hematopoietic cells such as myeloid, erythroid, and megakaryocytes series. Megakaryocytes decreased to a marked degree, which was surrounded by few platelets. Black, red, and green arrows represent myeloid cells, erythroid cells, and megakaryocyte, respectively. Arrow head represents platelets

A 60‐year‐old‐man with locally advanced NCSLC (cT3N1M0, stage IIIA) underwent concurrent chemoradiotherapy. He was treated with two cycles of cisplatin (60 mg/m^2^) plus tegafur‐gimeracil‐oteracil (120 mg/day) in combination with concurrent radiotherapy (60 Gy/30 fr). Irradiation was designed as shown in Figure 1. The irradiation area was close to the spinal cord, which might be associated with the myelosuppression. Although grade 3 neutropenia and thrombocytopenia required dose reduction in the second course, he completed the chemoradiotherapy. However, maintenance therapy with durvalumab was postponed owing to the sustained thrombocytopenia. He had not had hematologic diseases in the past. He had not taken any causal medication. Coagulation test showed hyperfibrinolysis through the entire course. A month later, durvalumab was initiated as the platelet count recovered to 142 × 10^3^/μL (Figure 2(a)). Soon after durvalumab was initiated, thrombocytopenia emerged at a minimum level of 55 × 10^3^/μL despite normal hemoglobin concentration and white cell counts. Durvalumab was discontinued after five cycles because thrombocytopenia did not ameliorate. Because the level of platelet‐associated IgG (PA‐IgG) was approximately twice as much as the normal level, bone marrow examination was conducted. The bone marrow was normoplastic and the pathology did not demonstrate cancer invasion, blastic proliferation, myelodysplasia, or myelofibrosis. Only the megakaryocyte depletion was demonstrated (Figure 3), which was inconsistent with that of idiopathic thrombocytopenic purpura (ITP). There were few platelets surrounding the megakaryocyte. In the bone marrow smear, nucleated cell counts were 56 000/μL, which indicated slight hypoplasia. The myeloid:erythroid (M:E) ratio was 1.48. The proportion of myeloid, erythroid, and lymphoid series were 53.2%, 32.6%, and 12.8%, respectively. We had not performed the bone marrow biopsy. He was treated with 30 mg of prednisolone, which was gradually tapered to 10 mg (Figure 2(b)). Six months later, the platelet counts recovered to the level of 110 × 10^3^/μL.

## DISCUSSION

Although ICIs are considered to be more tolerable than cytotoxic agent regarding hematological toxicities, thrombocytopenia can rarely be fatal. Up to today, there have been only approximately 10 reports.^4^ The diagnosis of immune‐mediated thrombocytopenia requires exclusion of other causes, such as dysplasia, cancer invasion, or coagulopathy. The patient's hyperfibrinolysis did not seem to affect thrombocytopenia because it did not change even after treatment. Furthermore, we also need to consider myelosuppression from chemoradiotherapy, especially in the setting of durvalumab maintenance therapy for locally advanced NSCLC. In the present case, we observed the elevated titer of PA‐IgG. Although PA‐IgG is not disease‐specific, it might imply the durvalumab‐mediated immune reaction. The exact mechanism of ICI‐mediated thrombocytopenia remains unknown. One of the hypotheses is that activation of CD4^+^ helper T cells and CD8^+^ cytotoxic T cells results in damaging the hematopoietic stem cells.^5^ Another is postulated to be platelet destruction by ICI‐induced production of platelet‐specific autoantibodies by preserved megakaryocytes in bone marrow, which is similar to the mechanism of ITP. The present case was inconsistent with typical ITP because the bone marrow specimen demonstrated the marked megakaryocyte depletion. Recently, acquired amegakaryocytic thrombocytopenic purpura (AATP) induced by ICI was reported.^6^ AATP is a rare bleeding disorder characterized by severe thrombocytopenia with preserved hematopoiesis of other lineages.^7^ Although the exact mechanism remains unclear, it is assumed that dysregulated humoral immunity against thrombopoietin (TPO), which potentiates platelet production by binding to the receptor on megakaryocytes, is involved.^8^ It was reported that serum TPO levels was quite high in patients with AATP whereas slightly high in those with ITP.^9^ Another case report demonstrated that the patients with AATP were seropositive for autoantibody against c‐mpl, which is a TPO receptor, and turned into seronegative with normal platelet count following immunosuppressant.^10^ It is important to distinguish ITP and AATP because corticosteroid or intravenous immunoglobulin is less effective in AATP than in ITP. In the present case, we measured neither serum TPO levels nor anti‐c‐mpl autoantibodies because there was no serum left before treatment. In addition, AATP could be the precursor for aplastic anemia,^11^ myelodysplastic syndromes (MDS),^12^ or acute leukemia.^13^ Although there is no standard treatment guideline for AATP, anecdotal episodes succeeding in the treatment with cyclosporine^13^ and TPO receptor agonist such as eltrombopag or romiplostim,^14^ were reported.

In conclusion, we demonstrated the first case of immune‐mediated thrombocytopenia during durvalumab maintenance therapy after chemoradiotherapy. We should call to mind not only the adverse effect of chemoradiotherapy, but also the immune‐mediated thrombocytopenia. Bone marrow biopsy should be considered in case of thrombocytopenia during maintenance treatment with an ICI.

## DISCLOSURE

The authors declare no conflicts of interest.

## INFORMED CONSENT

Written informed consent for the publication of clinical details were obtained from the patient.
